# PET image enhancement using artificial intelligence for better characterization of epilepsy lesions

**DOI:** 10.3389/fmed.2022.1042706

**Published:** 2022-11-16

**Authors:** Anthime Flaus, Tahya Deddah, Anthonin Reilhac, Nicolas De Leiris, Marc Janier, Ines Merida, Thomas Grenier, Colm J. McGinnity, Alexander Hammers, Carole Lartizien, Nicolas Costes

**Affiliations:** ^1^Department of Nuclear Medicine, Hospices Civils de Lyon, Lyon, France; ^2^Faculté de Médecine Lyon Est, Université Claude Bernard Lyon 1, Lyon, France; ^3^King's College London and Guy's and St Thomas' PET Centre, School of Biomedical Engineering and Imaging Sciences, King's College London, London, United Kingdom; ^4^Univ Lyon, INSA-Lyon, Université Claude Bernard Lyon 1, CNRS, INSERM, CREATIS UMR 5220, Lyon, France; ^5^Lyon Neuroscience Research Center, INSERM U1028/CNRS UMR5292, Lyon, France; ^6^CERMEP-Life Imaging, Lyon, France; ^7^Brain Health Imaging Centre, Center for Addiction and Mental Health (CAHMS), Toronto, ON, Canada; ^8^Departement of Nuclear Medicine, CHU Grenoble Alpes, University Grenoble Alpes, Grenoble, France; ^9^Laboratoire Radiopharmaceutiques Biocliniques, University Grenoble Alpes, INSERM, CHU Grenoble Alpes, Grenoble, France

**Keywords:** Monte-Carlo simulation, residual network, brain, focal cortical dysplasia (FCD), clinical application, deep learning, deblurring, super resolution (SR)

## Abstract

**Introduction:**

[^18^F]fluorodeoxyglucose ([^18^F]FDG) brain PET is used clinically to detect small areas of decreased uptake associated with epileptogenic lesions, e.g., Focal Cortical Dysplasias (FCD) but its performance is limited due to spatial resolution and low contrast. We aimed to develop a deep learning-based PET image enhancement method using simulated PET to improve lesion visualization.

**Methods:**

We created 210 numerical brain phantoms (MRI segmented into 9 regions) and assigned 10 different plausible activity values (e.g., GM/WM ratios) resulting in 2100 ground truth high quality (GT-HQ) PET phantoms. With a validated Monte-Carlo PET simulator, we then created 2100 simulated standard quality (S-SQ) [^18^F]FDG scans. We trained a ResNet on 80% of this dataset (10% used for validation) to learn the mapping between S-SQ and GT-HQ PET, outputting a predicted HQ (P-HQ) PET. For the remaining 10%, we assessed Peak Signal-to-Noise Ratio (PSNR), Structural Similarity Index Measure (SSIM), and Root Mean Squared Error (RMSE) against GT-HQ PET. For GM and WM, we computed recovery coefficients (RC) and coefficient of variation (COV). We also created lesioned GT-HQ phantoms, S-SQ PET and P-HQ PET with simulated small hypometabolic lesions characteristic of FCDs. We evaluated lesion detectability on S-SQ and P-HQ PET both visually and measuring the Relative Lesion Activity (RLA, measured activity in the reduced-activity ROI over the standard-activity ROI). Lastly, we applied our previously trained ResNet on 10 clinical epilepsy PETs to predict the corresponding HQ-PET and assessed image quality and confidence metrics.

**Results:**

Compared to S-SQ PET, P-HQ PET improved PNSR, SSIM and RMSE; significatively improved GM RCs (from 0.29 ± 0.03 to 0.79 ± 0.04) and WM RCs (from 0.49 ± 0.03 to 1 ± 0.05); mean COVs were not statistically different. Visual lesion detection improved from 38 to 75%, with average RLA decreasing from 0.83 ± 0.08 to 0.67 ± 0.14. Visual quality of P-HQ clinical PET improved as well as reader confidence.

**Conclusion:**

P-HQ PET showed improved image quality compared to S-SQ PET across several objective quantitative metrics and increased detectability of simulated lesions. In addition, the model generalized to clinical data. Further evaluation is required to study generalization of our method and to assess clinical performance in larger cohorts.

## Introduction

In the management of patients with epilepsy, approximately one third do not respond to medical therapy. For those with a focal onset, surgery could be their only potentially curative option (1). Identification of the epileptogenic zone (EZ), the zone where the seizure starts, is mandatory to allow planification of brain surgery. The EZ is the minimum brain tissue that needs to be resected to render the patient seizure-free, aiming at minimal functional impairment.

The presurgical evaluation workup includes history, semiology, EEG, video-EEG, and brain imaging (2). High-resolution brain magnetic resonance imaging (MRI) is the standard as it can identify structural lesions. However, in 35% of the cases, 3T MRI remains negative (3). In such cases, [^18^F]fluorodeoxyglucose ([^18^F]FDG) positron emission tomography (PET) can be used to improve EZ detection (4–6). The EZ appears as glucose hypometabolism (decreased FDG uptake) on interictal FDG-PET, particularly relevant in focal cortical dysplasia type 2 (FCD2) (7–9).

However, several degrading factors, including a low signal to noise ratio (SNR) and an intrinsically limited spatial resolution of PET scanners compromise PET image quality. The low resolution of PET images results in the partial volume effect (10) which leads to the spill-over of estimated activity across different regions (11). These alterations could falsely normalize or attenuate the relative hypometabolism of the EZ, notably when it is small (such as for FCDs), limiting the detection performance of PET (12, 13). The most commonly used approaches to address the noise (denoising) and resolution (deblurring) challenges are: (1) within-reconstruction methods such as early iteration termination of the reconstruction algorithm (14) or point spread function modeling (15, 16) and (2) post-reconstruction methods, such as gaussian filtering, but as this decreases the spatial resolution, many edge preserving alternatives were proposed (17–19). The most popular resolution recovery approaches in PET are partial volume correction (PVC) techniques but they rely on a segmented anatomical template based on MRI (20–23). Deconvolution methods that do not rely on structural information have also been proposed (24, 25). These methods partially correct the image but are still limited by the intrinsic resolution of PET physics and the statistical counting of the detection since they aim at converging to an explanatory distribution of the annihilation sites but not the emission sites of the positrons.

Artificial intelligence (AI)-based image enhancement is a very active field, but so far most of the publications focused on PET denoising rather than the deblurring problem (26). The deblurring problem involves the restoration of high-quality PET images (HQ) from lower-quality images [“standard quality (SQ)” PET images in our study] and not to restore a higher-count image from a low-count (low dose) PET image (denoising problem). Proof of concept of super-resolution PET has been validated with a 2D convolution neural network (CNN) in which the network was trained, using analytically simulated [^18^F]FDG PET, to predict their corresponding ground truth for normal brains (27) and lung tumors (28). This network is neither a simple deconvolution algorithm nor a partial volume correction algorithm. The aim of this project was to develop a deep learning based deblurring method consisting in predicting the ground truth from the PET image to improve epilepsy lesion visualization. Originality of the method was that the training was performed from simulated data, for which the ground truth is known. In order to improve clinical translation of such methods, we created a new, realistic set of [^18^F]FDG PET brain data using a validated Monte Carlo simulator (29–31) which were then reconstructed using Siemens e7 reconstruction tools. The 3D network trained to learn the mapping between the simulated SQ PET (S-SQ PET) and the corresponding ground-truth HQ (GT-HQ) PET did not require anatomical input. We assessed the quality of the network-predicted HQ (P-HQ) PET. We repeated the process for simulated lesional brain PET data with cortical focal hypometabolism to simulate difficult-to-detect small EZ. Lastly, we used real PET data to illustrate the proof-of-concept that a model trained on Monte-Carlo simulated PET data is applicable on real data.

## Materials and methods

### Medical image data

We used an open, multi-vendor [General Electrics, Philips and Siemens 3T magnetic resonance imaging (MRI) scanners] brain MRI database, Calgary-Campinas (32), using 173 T1-weighted (T1w) 3D volumes (1 mm^3^ voxels) from subjects with an average age of 53.4 ± 7.3 years (range 29–80, 50% women). Additionally, we used the publicly available database CERMEP-IDB-MRXFDG (33) which includes T1w MRI (Siemens 1.5T MRI) 3D volumes from 37 subjects (average age 38.11 ± 11.36 years; range 23–65, 54% women). It also includes 37 PET and computed tomography (CT) images from a Siemens Biograph mCT64, which we used to estimate a range of realistic FDG uptake values in brain PET as explained below. FDG PET data consisted in a static 10-min PET acquisition started 50 min after the injection of 122.3 ± 21.3 MBq of [^18^F]FDG. PET sinograms were reconstructed with Siemens' iterative ordered subset expectation maximization (OSEM) “High Definition” reconstruction, incorporating the spatially varying point spread function, with CT-based attenuation correction. To illustrate the capability of the developed AI deblurring method on clinical PET data, we used 10 datasets from epilepsy subjects with an average age of 23.3 ± 18.1 years (range 9–70, 50% women) acquired on the Siemens Biograph mMR at the King's College London and Guy's and St Thomas' PET Center, St Thomas' Hospital, London (Ethics Approval: 15/LO/0895). They consisted in a static 30-min PET acquisition started on average 120 ± 49 min after the injection of an average 120.6 ± 43.9 MBq of [^18^F]FDG.

### PET simulation

#### Generation of numerical brain phantoms

Numerical brain phantoms are 3D labeled volume models built from segmented T1w 3D volumes. We performed MRI non-parametric non-uniformity intensity normalization, tissue class segmentation, and anatomical parcellation of the T1w 3D volumes with Freesurfer (34). To expand the segmentation to extracerebral tissues, we also used SPM12 (35). We were then able to create an anatomical brain model with nine labels: gray matter (GM), white matter (WM) independently for the brain and the cerebellum (CEREB-WM, CEREB-GM), cerebrospinal fluid (CSF), basal ganglia (BG), bone, air, and soft tissue (SOFT).

#### Generation of ground truth high quality [^18^F]FDG PET

We created GT-HQ [^18^F]FDG PET by assigning activities to the nine labels of the numerical brain phantoms. Activities were derived from the distribution of normal [^18^F]FDG PET values from the CERMEP-iDB-MRXFDG database (33) after partial volume correction according to the Geometric Transfer Matrix (GTM) method (21).

We first simulated a series of normal brain SQ [^18^F]FDG PET scans. A total of 10 different brain activity distribution were generated for each anatomical brain model, resulting in 2100 (10 × 210) GT-HQ PETs. As a first step, WM activity was randomly chosen according to the observed distribution in (33). Activity ratios between cerebral GM and WM were then selected as 1.2, 1.8, 2.4, 3.0, 3.6, 4.2, 4.8, 5.4, 6.0, 6.6. Activities assigned to CSF, soft tissue and basal ganglia were randomly chosen according to the observed distribution in Mérida et al. (33). Cerebellum GM activity was set to 80% of the cerebrum.

Secondly, we created lesion GT-HQ PET phantoms with ROIs in the neocortex where we parametrically decreased assigned activity to simulate small metabolic lesions characteristic of FCDs. In 10 anatomical brain models with a GM/WM ratio of 3.6, we created one lesion each in the right frontal and in the left temporal region. The ROI for each lesion was manually defined as the largest component of the result of the multiplication of the GM mask and a sphere with volume of 1,008 mm^3^. In the same locations in the frontal and temporal lobes, we then repeated the process with two smaller spheres with volumes of 612 and 319 mm^3^. The resulting 60 lesion ROIs simulating small FCDs had volumes ranging from 17 to 570 mm^3^ with a mean of 184 ± 140 mm^3^: MRI volumetric values for FCDs ranged from 128 to 3,093 mm^3^ with a mean of 1,282 ± 852 mm^3^ (36). Activity ratios between cerebral GM and the lesion were assigned values of 0.6 and 0.3. This resulted in 60 (10 models × 3 sizes × 2 activity ratios) lesion GT-HQ PET (120 lesions) with various morphologies and activities.

#### Monte-Carlo simulation of standard-quality PET

To generate realistic PET acquisitions, we used SORTEO, a Monte Carlo PET simulator developed by Reilhac et al. (31) and validated to provide realistic simulations for the Siemens Biograph mMR scanner (29, 31). The simulated 3D emission protocol consisted in the collection of data into a single timeframe for a 30-min period, as in our institution, starting 40 min post-injection, in accordance with international FDG PET guidelines (37). SORTEO generates the sinogram (raw data), by simulating each disintegration occurring in labels where a constant activity was defined (GM, WM, CSF, CEREB-WM, CEREB-GM, GN and SOFT) including all physical phenomenon occurring from positron emission to detection. As for clinical scans, sinograms were normalized and corrected for randoms, scatter, attenuation, dead-time, and radioelement decay.

The simulations were performed at the IN2P3 (CNRS UAR6402) computing center. For each subject, simulation was divided into eight sub-processes to take advantage of multi-core processing and thus reducing the total simulation time.

#### Tomographic reconstruction

Corrected simulated sinograms were reconstructed with e7 reconstruction tool™ (Siemens Healthineers) using a 3D ordinary poisson-ordered subsets expectation maximization algorithm, incorporating the system point spread function, using 3 iterations and 21 subsets. Reconstructions were performed with a matrix size of 172 × 172 × 127 and a zoom factor of 2, yielding a voxel size of 2.04 × 2.04 × 2.03 mm^3^. The attenuation correction used a pseudo-CT synthetized with MaxProb multi-atlas attenuation correction method from the T1w MRI (38). Gaussian post-reconstruction 3D filtering (FWHM 4 mm isotropic) was applied to all PET images.

In the end, we have a database of 2100 pairs of GT-HQ and S-SQ PET images, with various anatomies and activity contrasts between brain structures. In addition, we simulated 120 small metabolic lesions characteristic of FCDs with various morphologies and activities.

### Deep learning

#### Residual network architecture

**Figure 1:**
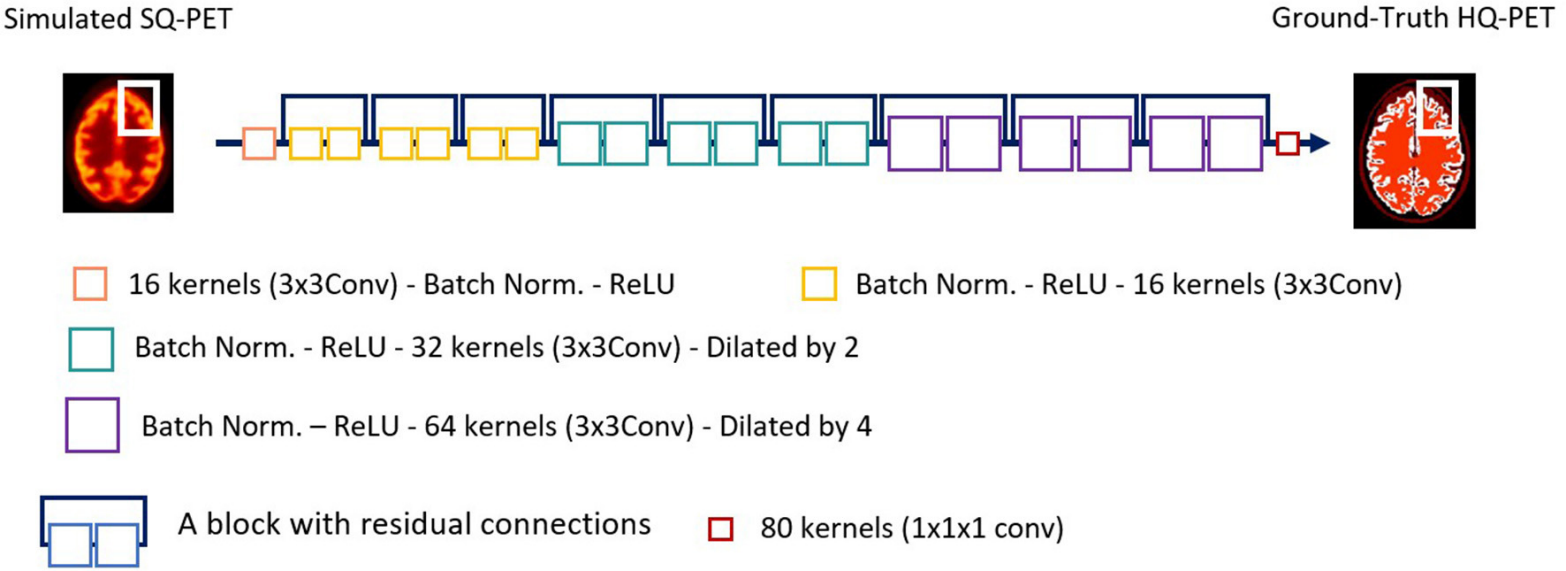
Architecture of the ResNet network used in this study. Conv, convolution; ReLU, Rectified Linear Unit; PET, Positron Emission Tomography; SQ, standard-quality; Norm, normalization; HQ, High-quality.

Residual CNNs are commonly used algorithms for PET deblurring and are the main algorithms used for the generator in generative adversarial networks (26). The proof-of-concept of super-resolution PET was based on a very deep CNN (20 layers) (27) which was 2D because of computation limitation. As 3D images proved more successful in denoising tasks (39), we developed a 3D network for super resolution PET. Initially, we used a 3D U-Net, the main 3D network implemented for denoising PET (26). However, 3D U-Net did not achieve satisfactory results for our task and so we used a 3D sequential ResNet (40), similarly to recent papers by Spuhler et al. (41) and Sanaat et al. (42) with dilated kernels (model comparison shown in Supplementary material). They enlarge the field-of-view to incorporate multiscale context (43–45) and avoid the up-sampling layers of U-Net that degrade resolution, as spatial resolution of the input is maintained throughout the network (42). We implemented the model shown in Figure 1. Each of the first 19 modules of the network exclusively uses convolutional kernels of size 3 × 3 × 3, along with batch normalization and Rectified Linear Unit (ReLU) activation function. In the first 7 modules, the network uses 16 kernels, the following 6 modules use 32 kernels, but with a dilation parameter of 2, and the next 6 modules use 64 kernels with dilation 4. The input of the deep learning model is the S-SQ PET.

#### Data preprocessing

Trilinear interpolation was used to resample all PET images to the same voxel size of 1 × 1 × 1 mm with a 192 × 256 × 256 grid size. The intensities in the input S-SQ PET images were standardized by dividing by the average of each individual image. Each GT-HQ PET was standardized by the average of the corresponding S-SQ PET image. The standardization factors were stored and subsequently applied to the network's predictions to rescale the resulting images, before performing any quantitative analysis [the PET unit was Becquerel (Bq) per: *centimetres cubed* (cm^3^)].

#### Network implementation and optimization

The simulated images were split into training, validation, and testing datasets, with a ratio of 80/10/10%. Due to limitations of GPU memory during training, the network was trained with 32 × 32 × 32 voxel patches. Twenty patches per volume were randomly chosen for the training and validation set. Mean absolute error was used as the loss function during training and the optimizer was AdamW (46). The learning rate was set to 10^−4^ and reduced by a factor of 0.1 when the validation loss stagnated for more than 10 epochs. The batch size was set to 50 and the maximum number of epochs to 200 using early stopping (validation loss not improving during more than 60 epochs).

We trained our model on a GPU server on 1 NVIDIA V100 GPU (32GB) running Python 3.9.10, Pytorch 1.10.0 (47), and TorchIO 0.18.71 (48).

For inference, patch of size 32 × 32 × 32 voxels were used with 8 × 8 × 8 overlapping tile stride. These patches were selected in sequence from the whole 192 × 256 × 256 volume, then the P-HQ patches were put together to generate the entire P-HQ PET. Overlapping patches were combined using a weighted averaging strategy.

### Evaluation

Evaluation of AI-enabled super-resolution PET was carried out on the P-HQ PET by comparing it to the S-SQ PET and the GT-HQ PET in brain masked images. We used the following quantitative evaluation metrics: (1) the Peak Signal-to-Noise Ratio (PSNR) (49), (2) the structural similarity index measure (SSIM) (50) which is a well-accepted measure of perceived image quality *s*, and (3) the root mean squared error (RMSE) (Equations 1–3, respectively). An objective improvement in image quality is reflected by larger values in peak signal to noise ratio (PSNR) and structural similarity index metrics (SSIM) and smaller values for the root mean square error (RMSE).


(1) $$\begin{array}{l} PSNR\;\left(X,Y\right)=20\;\times \;\log_{10}^{\left(\;\frac{Max\left(X\right)}{\sqrt{MSE\left(X,Y\right)}}\right)} \end{array}$$

(2) $$\begin{array}{l} SSIM\;\left(X,Y\right)=\;\frac{\left(2\mu_{x}\mu_{y}+c_{1}\right)\left(2\sigma_{xy}+c_{2}\right)}{\left(\mu_{x}^{2}+\mu_{y}^{2}+c_{1}\right)\left(\sigma_{x}^{2}+\sigma_{y}^{2}+c_{2}\right)} \end{array}$$

(3) $$\begin{array}{l} RMSE\;\left(X,Y\right)=\sqrt{\frac{\overset{L}{\underset{j=1}{\sum }}{\left(X-Y\right)}^{2}}{L}} \end{array}$$

In Equation (1), given two images X and Y, *Max*(X) indicates the maximum intensity value of X, whereas MSE is the mean squared error. In Equation (2), μ_x_ and μ_y_ denote the mean value of *X* and *Y*, respectively. σ_xy_ indicates the covariance of σ_x_ and σ_y_, which in turn represent the variances of *X* and *Y*, respectively. The constant parameters c_1_ and c_2_ (c_1_ = 0.01 *and* c_2_ = 0.03) were used to avoid a division by very small numbers. In Equation (3), *L* is the total number of voxels in the head region, *X* and *Y* are the two compared images.

For the next evaluations, we used GM and WM ROIs, issued form the GM and WM probability maps resulting from T1w MRI segmentation using Freesurfer as described in 2.2.1. The WM ROI was obtained from the WM mask eroded by a radius of 6 voxels using ITK (51) to give a conservative WM ROI. The mean GM ROI volumes were 948,106 ± 102,640 mm^3^ and the mean eroded WM ROI volumes were 486,509 ± 63,909 mm^3^.

Recovery coefficients (RCs) defined as the ratio between the observed activity and the ground truth activity as shown in Equation (4), were calculated using μ the mean value in the GM ROI and the WM ROI for S-SQ PET and P-HQ PET compared to the GT-HQ PET.


(4) $$\begin{array}{l} RC_{mean}=\;\frac{{\mu \;}_{measured}}{{\mu \;}_{ground\;truth}} \end{array}$$

We also computed the coefficient of variation (CoV) defined as the ratio between σ, the standard deviation, and μ, the mean value in the ROI, as shown in Equation (5). It is a metric for describing ensemble noise or statistical noise and it was computed in the GM ROI and the WM ROI for S-SQ PET and P-HQ PET.


(5) $$\begin{array}{l} COV=\;\frac{\;\sigma_{measured}}{{\;\mu }_{measured}}\times 100 \end{array}$$

For lesion assessment, we performed first a visual assessment. The reader evaluated two sets of PET images: P-HQ PET images and S-SQ images in a random order. The reader determined whether a hypometabolic lesion was present (0 = none, 1 = visible lesion), and scored overall diagnostic confidence (ODC) in interpreting the images on a Likert scale of 1–5 (1 = none, 2 = poor, 3 = acceptable, 4 = good, 5 = excellent diagnostic confidence) (52) for each lesion. A second reader performed a visual assessment of a subset of lesioned S-SQ PET and G-HQ PET (*n* = 84 images) to assess inter-reader concordance. Secondly, to quantify lesion detectability, we computed a ratio between the measured activity in the ROI of the lesion over the same ROI in the P-HQ PET image without the lesion, termed relative lesion activity (RLA). We also computed the recovery coefficient in the lesion as in Equation (4).

For clinical data, we performed a visual assessment of the clinical PET and the P-HQ clinical PET computed with the trained network (*n* = 20 images) by two readers. The reader scored the diagnostic image quality on a 5-point Likert scale (1 = non-diagnostic, 2 = poor, 3 = standard, 4 = good, 5 = excellent image quality) (52) and as previously, indicated if a hypometabolic lesion was present and scored ODC.

We compared the quantitative results through the different metrics with pairwise *t*-tests or Wilcoxon rank sum test. Kappa coefficients were computed to assess inter-reader agreement. For all comparisons, the threshold of statistical significance was set at 5%.

## Results

### Non-lesioned simulated brains

**Figure 2:**
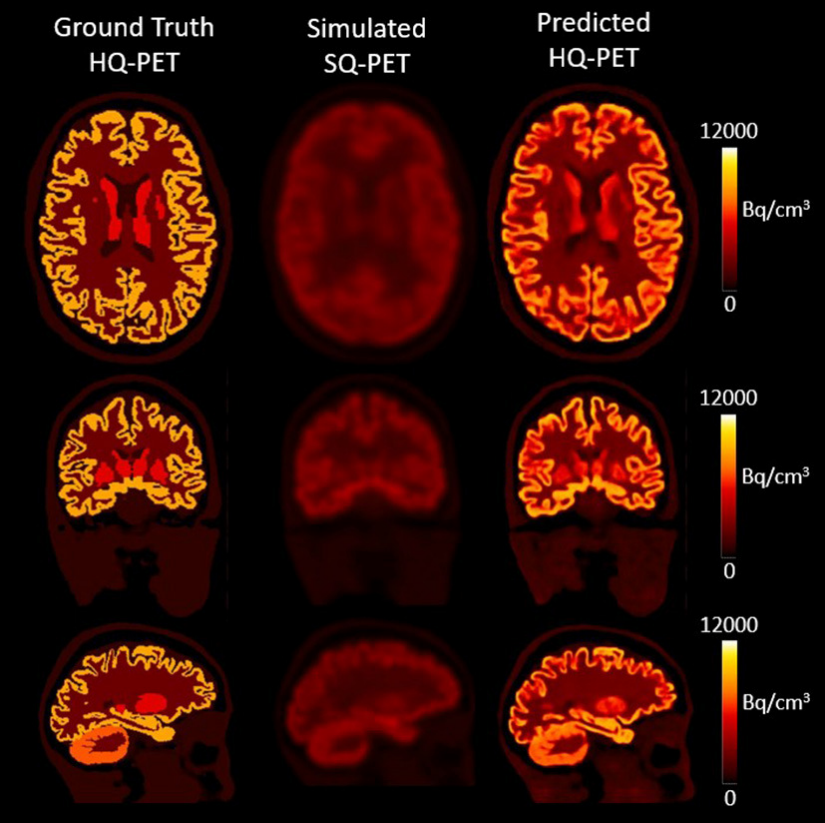
Results from one subject belonging to the test dataset. The first column depicts the Ground Truth High Quality (HQ) PET, the second column the corresponding simulated Standard Quality (SQ) PET and the third column the Predicted HQ PET, i.e., the output from the proposed network. For each set, from top to bottom, transverse, coronal, and sagittal slices are shown. Images are displayed using radiological conventions (subject's left on the right). Bq, Becquerel.

The model was successfully trained to learn the mapping from the S-SQ PET to the GT-HQ PET after 105 epochs. Figure 2 showcases the result for one subject from the test dataset in transverse, coronal, and sagittal slices for the GT-HQ PET, its corresponding S-SQ PET, and the P-HQ PET.

**Figure 3:**
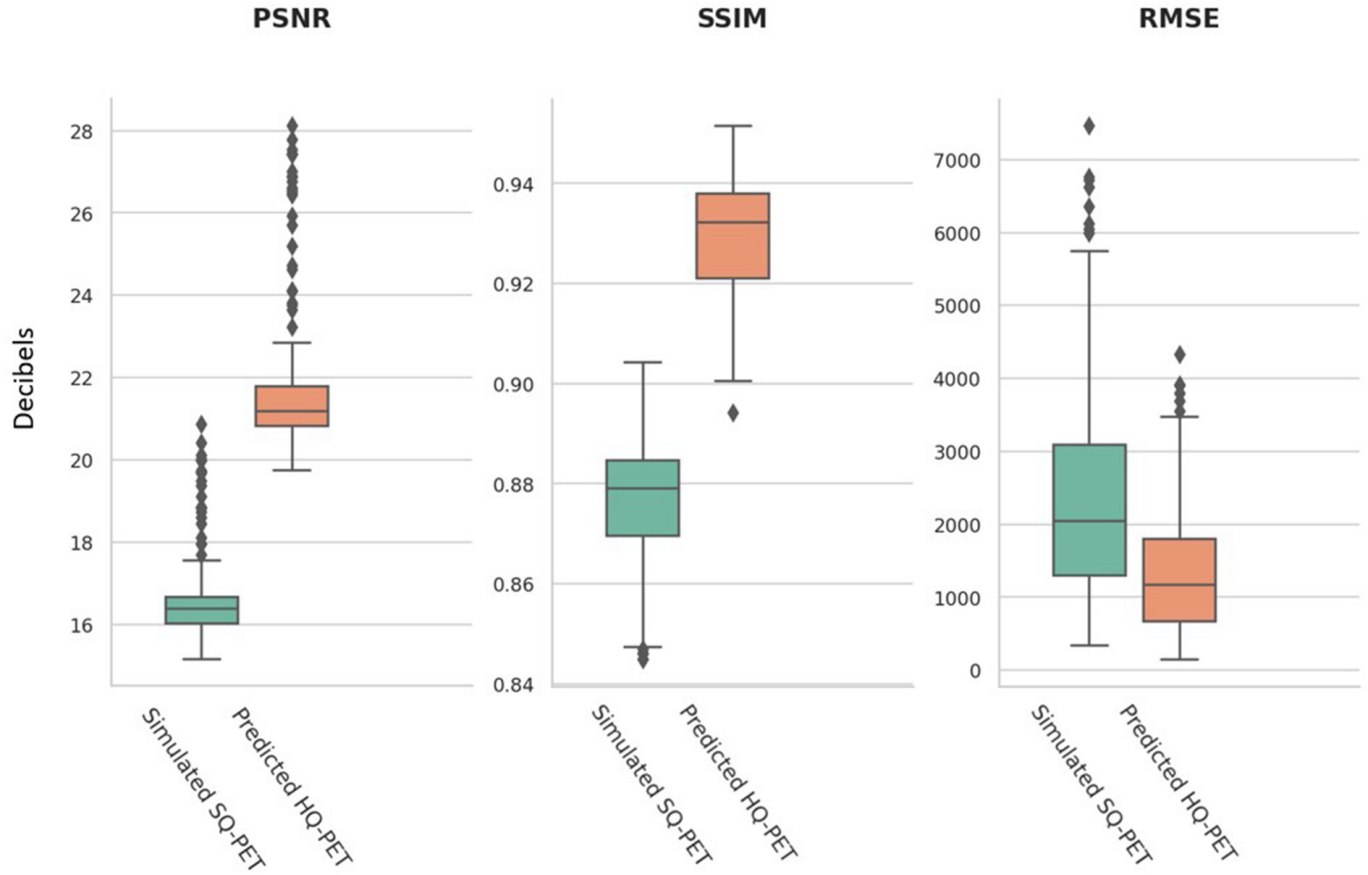
Image quality metrics from the simulated standard-quality (SQ) PET and the predicted high-quality (HQ) PET for the test set. An objective improvement in image quality is reflected by larger values in peak signal to noise ratio (PSNR) and structural similarity index metrics (SSIM) and smaller values for the root mean square error (RMSE).

The performance metrics computed on the test set for the P-HQ PET are shown in Table 1 and are plotted in Figure 3. The values of those metrics on the S-SQ PET were also included for comparison. P-HQ PET showed improved image quality compared to the S-SQ PET (*p* < 0.0001 for all comparisons).

**Table 1:** Mean and standard deviation of the root mean squared error (RMSE), peak signal to noise ratio (PSNR), and structural similarity index measure (SSIM) for simulated standard quality and predicted high quality (HQ) PET images in the test set.

	**Root mean squared error**	**Peak signal-to-noise ratio (dB)**	**Structural similarity index measure**
Simulated Standard-quality PET	2,393 ± 1,496	16.6 ± 1.1	0.876 ± 0.013
Predicted High-quality PET	1,359 ± 888	21.8 ± 1.8	0.929 ± 0.011

The comparator is the ground-truth HQ PET.

We computed the recovery coefficient of the GM and the WM in the test set for the S-SQ PET and the P-HQ PET. Recovery coefficients were significantly improved in the P-HQ PET for the WM and the GM compared to the S-SQ PET (*p* ≤ 0.0001). Mean, standard deviation (SD) and range of the recovery coefficient (RC) for the gray matter and the white matter for P-HQ-PET and S-SQ PET in the test set are shown in Table 2.

**Table 2:** Mean, standard deviation (SD) and range of the recovery coefficient (RC) for the gray matter (GM) and the white matter (WM) for predicted high-quality (HQ) PET and simulated standard-quality (SQ) PET in the test set.

	**Simulated SQ PET**		**Predicted HQ PET**	
	**GM RC**	**WM RC**	**GM RC**	**WM RC**
Mean ± SD	0.29 ± 0.03	0.49 ± 0.03	0.79 ± 0.04	1 ± 0.05
Range	0.22–0.38	0.35–0.56	0.65–0.94	0.8–1.14

We further analyzed by GM/WM ratios (Boxplots shown in Supplementary Figures 2, 3). For all GM/WM ratios, GM recovery as well as WM recovery were significantly improved for the P-HQ compared to the S-SQ PET (*p* < 0.0001). In the S-SQ PET, across all GM/WM ratio, the mean GM RC ranged from 0.26 to 0.36 and standard deviation ranged from 0.008 to 0.220; the WM RC ranged from 0.45 to 0.52 and standard deviation range from 0.008 to 0.044. For the P HQ-PET, the mean GM RC ranged from 0.77 to 0.86 and standard deviation range from 0.016 to 0.042 and for the WM RC, it ranged from 0.99 to 1.04 and standard deviation ranged from 0.024 to 0.047. *Post-hoc* Anova analysis showed a significative difference for GM and WM RC, with a better RC for the lowest ratio (1.2).

The mean COV across all test datasets in the GM ROI was 38.9 ± 2.0 in the S-SQ PET and minimally higher at 39.3 ± 2.0 in the P-HQ PET (difference not significant, *p* = 0.051). The mean COV in the WM ROI was very similar at 4.90 ± 0.89 for S-SQ PET and 4.91 ± 0.89 for P-HQ PET (*p* = 0.97).

### Lesioned simulated brain

At the group level, the visual detection rate was 38% in the S-SQ PET increasing to 75% in the P-HQ PET (*p* < 0.05) with a similar overall diagnostic confidence score of 3.3 ± 1.6 vs. 3.5 ± 1.5 (*p* > 0.05). Kappa coefficients for inter-reader concordance were 0.77 for all images, 0.88 for P-HQ PET and 0.72 for S-SQ PET. Overall mean visual detection rates (44 vs. 42% in the S-SQ PET and 75 vs. 72% in the P-HQ PET) and diagnostic confidence scores (3.2 ± 1.7 vs. 3.1 ± 1.5 in the S-SQ PET and 3.4 ± 1.5 vs. 3.5 ± 1.3 in the P-HQ PET) were not statistically different between readers.

**Figure 4:**
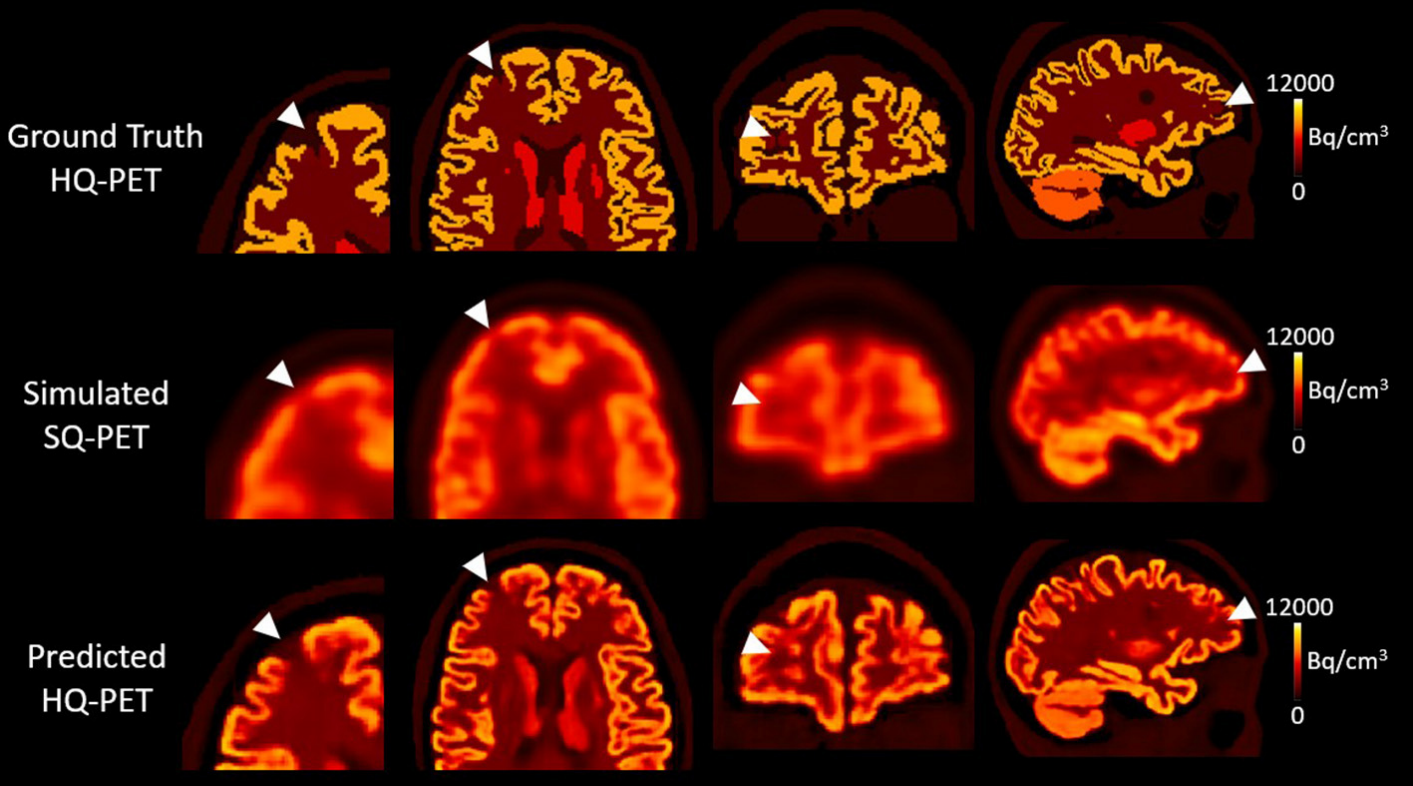
Results from one subject belonging to the test dataset with a simulated right frontal hypometabolic lesion with a volume of 0.327 cm^3^. First column, enlarged view of the lesion in transverse view; second column, transverse view, third column, coronal view, fourth column, sagittal view. The relative lesion activity was 0.3 in the ground-truth high-quality-PET, 0.75 in the simulated standard-quality PET, and decreased to 0.44 in the predicted HQ-PET. Arrowheads indicate the location of the simulated lesion. Images are displayed using radiological conventions (subject's left on the right). Bq, Becquerel *cm*^3^*: centimetres cubed*.

Figure 4 shows an example of one subject with a right frontal hypometabolic lesion of 327 mm^3^ from the test dataset for the GT-HQ PET, the S-SQ PET and the P-HQ PET. Through visual inspection, the hypometabolic lesion was easier to detect and with more confidence on the P-HQ PET. The RLA was 0.75 in the S-SQ PET, decreasing to 0.44 in the P-HQ PET, closer to the ground truth of 0.3.

**Figure 5:**
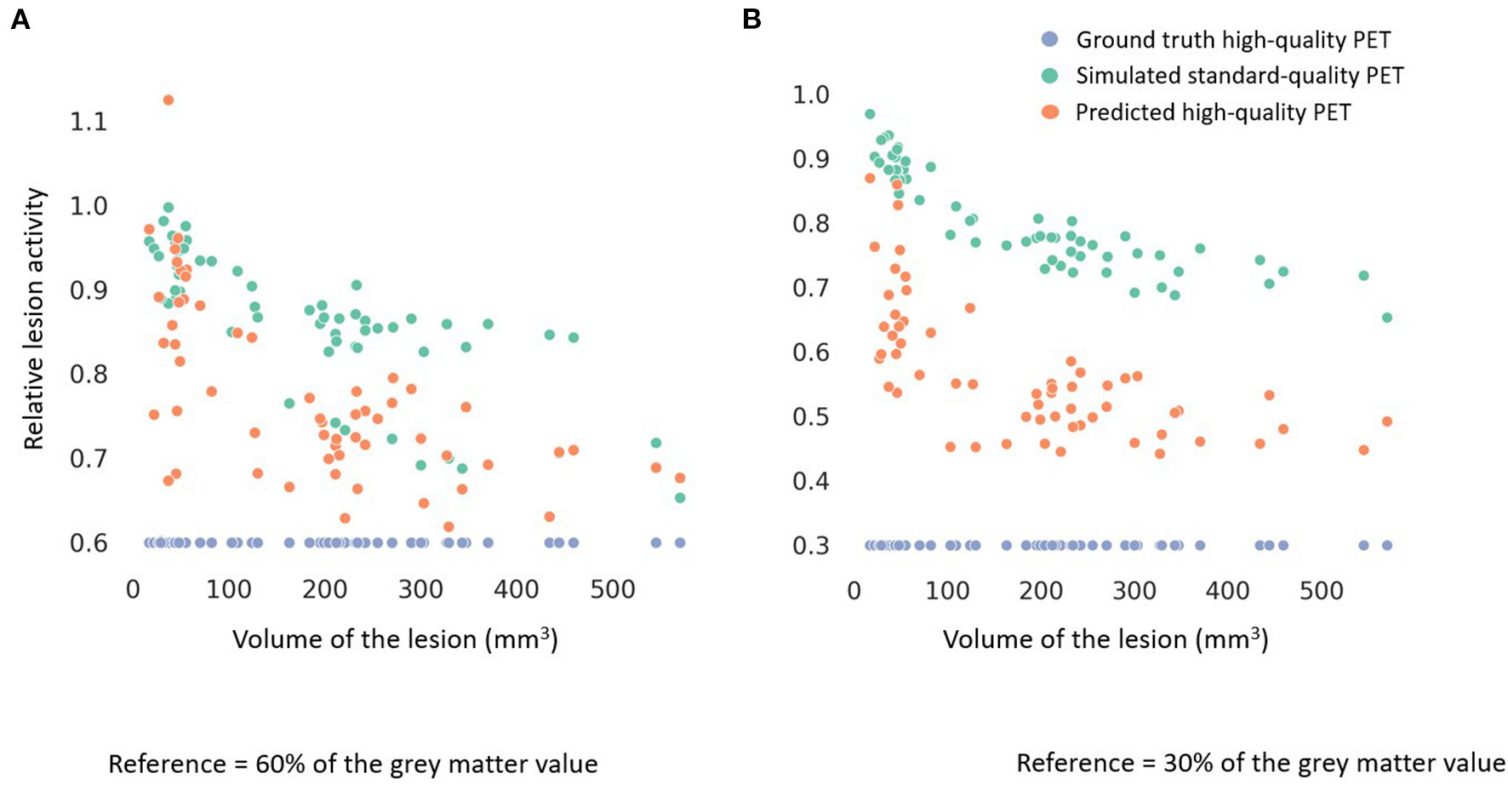
Scatter plots of the relative lesion activity in the ground truth high-quality (HQ) PET (blue dot), the simulated standard-quality (SQ) PET (green dot) and the predicted HQ PET (orange dot) according to the volume of the lesions (mm^3^). The lesion ground truth activity in **(A)** was 60% of the gray matter normal activity and in **(B)** was 30% of the gray matter normal activity.

**Figure 6:**
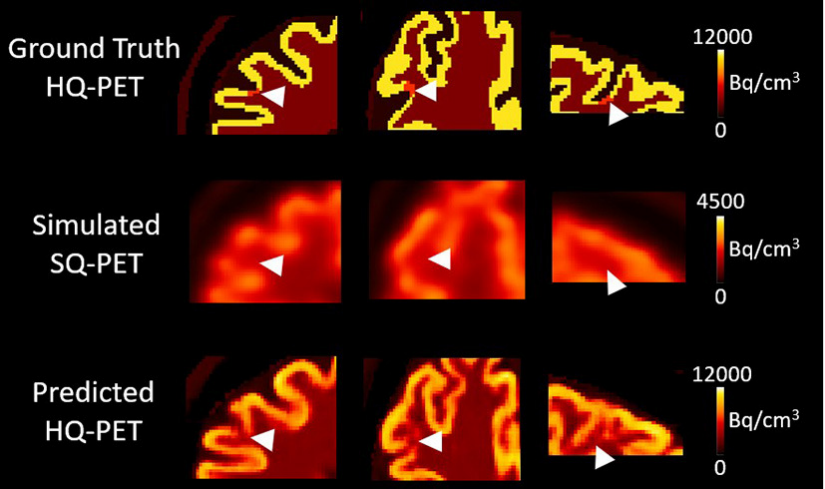
Results from one subject belonging to the test dataset with a simulated right frontal hypometabolic lesion with a volume of 22 mm^3^. First column, transverse view, second column, coronal view, third column, sagittal view centered on the lesion. The relative lesion activity was 0.6 in the ground-truth high-quality (HQ) PET, 0.95 in the simulated standard-quality (SQ) PET, and decreased to 0.75 in the predicted HQ PET. Arrowheads indicate the location of the simulated lesion. Images are displayed using radiological conventions (subject's left on the right). Bq, Becquerel *cm*^3^*: centimetres cubed*.

Among all the lesions (GT-HQ PET RLA 0.3 or 0.6), RLA was substantially higher at 0.83 ± 0.08 (0.65–1) in the S-SQ PET but decreased toward the GT-HQ PET with 0.67 ± 0.14 (0.44–1.12) (*p* < 0.0001) in the P-HQ PET. RLA according to lesion volumes (mm^3^) are plotted in Figure 5 for both the ground truths set at 0.3 or 0.6. There is a negative relation between the size of the lesion and the RLA value. For each subgroup whose GT-HQ PET RLA was 0.3 or 0.6, mean RLA, relative RLA error and RC and their standard deviations are presented in Table 3. For the subgroup whose GT-HQ PET RLA was 0.3 (high contrast between lesion and surrounding GM), the mean RLA value for S-SQ PET was 0.80 ± 0.08 (0.65–0.97) and decreased to 0.57 ± 0.11 (0.44–0.87) in P-HQ PET. Values were significantly lower in the P-HQ PET (*p* < 0.0001) but remained significantly superior to the GT-HQ PET RLA of 0.3 (*p* < 0.0001). The mean relative RLA error in the S-SQ PET was 1.68 ± 0.26 (1.18–2.23) vs. 0.89 ± 0.35 (0.47–1.90) in P-HQ PET (*0* < 0.0001). The mean RC in the lesion ROI was 0.71 ± 0.10 (0.55–0.97) for the S-SQ PET vs. 1.44 ± 0.33 (1.05–2.5) for the P-HQ PET (*p* < 0.0001). For the subgroup whose GT-HQ PET RLA was 0.6 (low contrast between lesion and surrounding GM), the mean RLA value for the S-SQ PET was 0.86 ± 0.08 (0.65–1) and decreased to 0.77 ± 0.11 (0.60–1.12) in P-HQ-PET. Values were significantly lower in the P-HQ PET (*p* < 0.0001) but remained significantly superior to the GT-HQ PET RLA of 0.6 (*p* < 0.0001). The mean relative RLA error for the S-SQ PET was 0.44 ± 0.17 (0.09–0.66) vs. 0.28 ± 0.18 (0.00–0.87) in the P-HQ PET (*p* < 0.0001). Finally, the mean RC in the lesion ROI was 0.39 ± 0.05 (0.32–0.51) for the S-SQ PET vs. 0.98 ± 0.17 (0.69–1.45) for the P-HQ PET (*p* < 0.0001). Mean RC in P-HQ PET and GT-HQ PET were not different (*p* = 0.32). In Figure 6, we show a small lesion in the frontal lobe. The RLA was 0.6 in the GT-HQ PET, 0.95 in the S-SQ PET, and decreased to 0.75 in the P-HQ PET.

**Table 3:** Mean and standard deviation of the lesion relative lesion activity (RLA), the relative RLA error, and the lesion recovery coefficient (RC) for simulated standard quality, and predicted high quality (HQ) PET images in the test set.

	**GT-HQ PET RLA lesion** = **0.3**			**GT-HQ PET RLA lesion** = **0.6**		
	**Lesion RLA (Target: 0.3)**	**Relative RLA error** **(Target: low)**	**Lesion RC (Target: 1)**	**Lesion RLA** **(Target: 0.6)**	**Relative RLA error (Target: low)**	**Lesion RC** **(Target: 1)**
Simulated standard-quality PET	0.80 ± 0.08	1.68 ± 0.26	0.71 ± 0.10	0.86 ± 0.08	0.44 ± 0.17	0.39 ± 0.05
Predicted high-quality PET	0.57 ± 0.11	0.89 ± 0.35	1.44 ± 0.33	0.77 ± 0.11	0.28 ± 0.18	0.98 ± 0.17

The comparator is the ground-truth (GT) HQ PET. Left panel, lesion RLA was 0.3; right panel, lesion RLA was 0.6.

### Epilepsy patients

**Figure 7:**
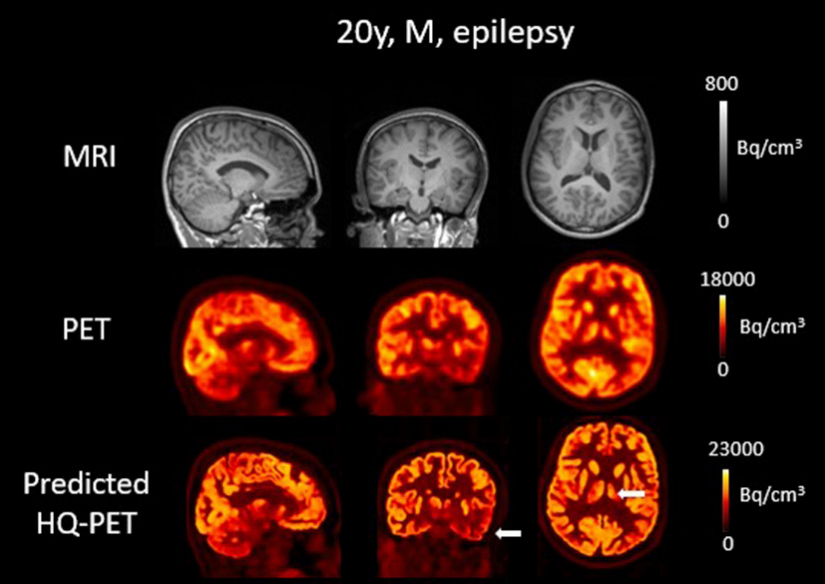
Brain T1w MRI and clinical [^18^F]FDG PET as well as predicted high-quality (HQ) PET (predicted by the network developed in this work) from one patient with drug-resistant epilepsy. Images are displayed using radiological conventions (subject's left on the right) and white arrows are used to highlight areas of hypometabolism. The first two rows show images from the scanner and the third row shows the AI-enhanced high-quality PET. There was no clear anomaly on the MR but a hypometabolism in the left temporal lobe as well as in the left thalamus on both PET images. Bq, Becquerel *cm*^3^*: centimetres cubed*.

**Figure 8:**
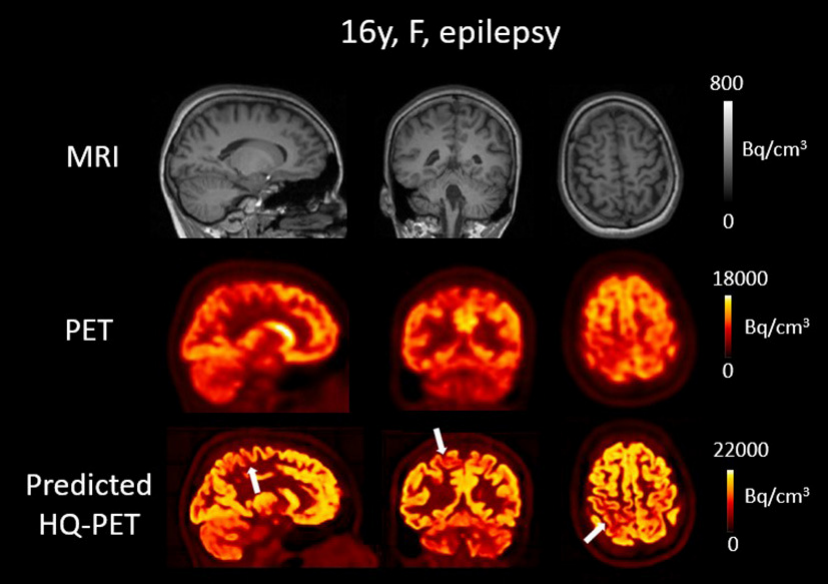
Brain T1w MRI and clinical [^18^F]FDG PET as well as predicted high-quality (HQ) PET (predicted by the network developed in this work) from one patient with drug-resistant epilepsy. Images are displayed using radiological conventions (subject's left on the right) and white arrows are used to highlight areas of hypometabolism. The first two rows show images from the scanner and the third row shows the AI-enhanced high-quality PET. MRI depicted a blurred white matter gray matter border in the right postcentral gyrus. The PET showed a correlated blurred and mild hypometabolism extending toward the precuneus. The predicted HQ PET showed a clearer hypometabolism very well correlated with the lesion that also extended to the precuneus. Bq, Becquerel *cm*^3^*: centimetres cubed*.

The result of the trained model for clinical data is illustrated in Figures 7, 8 showing brain T1w MRI, [^18^F]FDG PET and the P-HQ PET from two different patients with drug-resistant epilepsy. Across the cohort of epilepsy patients, the mean diagnostic image quality ratings for the clinical PETs were 2.9 ± 0.3 vs. 3.9 ± 0.5 for the predicted HQ PET (*p* < 0.01). Inter-reader mean quality scores were not significantly different. The mean diagnostic confidence ratings were 3.4 ± 1.1 for the clinical PET vs. 4.2 ± 0.8 for the predicted HQ (*p* = 0.02). Inter-reader mean confidence rating scores were not significantly different. Lesion detection rates were identical for both readers (7/10) for both the clinical PET and the predicted HQ PET.

## Discussion

In this work, we trained a network to map Monte-Carlo S-SQ PET to their GT-HQ PET. In an independent test set, the P-HQ PET showed improved image quality compared to S-SQ PET across several objective quantitative metrics. In an independent dataset with small, simulated epilepsy lesions, the P-HQ PET significantly improved the relative lesion activity and visual detectability. Lastly, we have shown that the model was able to generalize to clinical data, illustrating the proof-of-concept that a model trained on Monte-Carlo simulated PET data is applicable on real data.

To train our model we had to overcome the limited availability of high quality training data, a common challenge for the deblurring problem (53) and so we chose to use simulation. We developed a pipeline based on a Monte-Carlo based PET simulator as it can accurately model the PET acquisition process including physical effects resulting in realistic sinograms (29) that have the same data distribution as the real PET. Compared to the few papers about PET deblurring with AI in image space, our simulated PET were more realistic: two studies used physically unrealistic degradation methods for their S-SQ PET adding Gaussian noise to an inverted T1w MR or down-sampling the standard PET image (54, 55). The latter approach also does not allow improvement beyond S-SQ PET. Two other studies used PET simulated analytically rather than with a Monte Carlo method (27, 28). While the main drawback of Monte-Carlo simulation is the computational burden, we were able to simulate PET acquisitions in a reasonable amount of time (about 3 h per scan) using PET SORTEO (29) which has been validated to provide realistic simulation of the Siemens Biograph mMR PET-MR (31), a system available across both our institutions. To be as close as possible to the clinical PET images, we reconstructed the generated sinogram using e7tools™ (Siemens Healthineers), which is also used for clinical data. Next, we used the same pipeline to generate data with a simulated epileptogenic lesion. This pipeline now enables creation of a whole range of realistic datasets for training if needed.

Our model has several particularities. We went beyond previous published PET deblurring methods with AI which used 2D models (27, 28, 54–56). We developed a 3D model, following results in the PET denoising field where 3D models tend to outperform 2D or 2.5D models (39, 57) because of additional features in 3D space. To prevent the impact of regional homogeneity on GT-HQ PET on the model parameters, we trained using small brain patches (32 × 32 × 32 mm^3^) from PET data simulated with of large number of GM/WM ratios. Thus, at the end of the training, the network weights were defined to respond to a wide range of voxel values (including hypometabolism) and patterns. The inference was also computed using the same size of patches which were then put together to obtain the predicted P-HQ PET. Compared to many deblurring methods (including some PVC methods and AI-based approaches) which rely on anatomical information (26), we provide a model that only relies on PET data which offers multiples advantages. Firstly, as the method works in the image space it can be applied on previously acquired PET even if raw data (sinograms) are not or no longer available, as will be the case in most clinical centers. Secondly, with the current development of dedicated standalone brain PET scanners (58), a PET-only method offers a unique opportunity to be combined with novel high-performance, high-resolution hardware to detect very small lesions. Thirdly, using a PET-only method prevents potential performance degradation that could stem from inter-modality alignment errors (59) which can occur even with simultaneous PET-MR if the MR sequence used for deblurring has been acquired at a different time to the emission data under study.

Our model achieved very good performance for relative lesion activity, which depicts lesion contrast, among all the lesions, despite different localization or shape. In the S-SQ PET, RLA was substantially higher at 0.83 ± 0.08 but decreased toward the GT-HQ PET ground truth (0.45) with 0.67 ± 0.14 in P-HQ PET. There was one outlier in the 0.6 RLA group with a P-HQ PET RLA value above one for a 37 mm^3^ lesion in the lateral temporal lobe. This occurred because in the S-SQ PET, the lesion had a RLA value (0.997) so close to 1 that the information about presence of a lesion was lost during the simulation process. This is a principal limitation of our model which will only be overcome with higher resolution hardware. However, these results suggested that the model improved the RLA for most lesions even largely inferior to the nominal average 1D spatial resolution of 4.3 mm in full width at half maximum of the Siemens Biograph mMR (60), which defined a volumetric resolution near 80 mm^3^. The quantitative results correlated well to the visual analysis of the P-HQ PET images showing increased visibility of the simulated lesions as well as slight improvement in the confidence of the reader, suggesting the improvements from P-HQ PET are relevant for future clinical application for epilepsy presurgical PET assessment.

Even if the SORTEO simulator is validated for the PET-MR, clinical PET images from the PET-MR will be slightly different requiring normalization, so the clinical P-HQ PET was expected to be different. Nevertheless, distributions of the simulated data and real data were close enough to enable use of both data types with the same network. We therefore consider that the clinical data application was successful in illustrating proof-of-concept that a model trained on Monte-Carlo simulated PET data is applicable on real data. Whereas, generalizability to out-of-distribution data is a common critical limiting factor for deep learning-based image processing (53), in our case this limitation could in principle be overcome by creating more simulations using the Monte-Carlo pipeline with settings tuned (29, 31) to simulate different scanners and reconstructions. Nevertheless, a study of generalization exceeds the scope of this manuscript. Such a study would need to be carefully planned to include reconstruction methods, scanner manufacturer, injection dose, uptake time and acquisition time to quantify the potential of such methods and their limits.

The realistic Monte-Carlo PET simulations and our training method allowed us to directly apply the trained network on clinical data. The P-HQ PET of the patients again showed an improved visual quality as well as an improved reader confidence. When we visually compared the GT-HQ PET and the P-HQ PET, it was apparent that the cortical structures were similar indicating that P-HQ PET from clinical data should not mislead physicians. Also, the clinical reading of epilepsy imaging does not rely on PET only. Indeed, physicians are trained to read both PET and MRI independently first, and jointly later, using the additional information to interpret the metabolism. In addition, and as in clinical practice (for example with non-attenuation corrected images), the non-enhanced standard quality image would always be made available to the reading physician to consult. One limitation of the clinical application was the small retrospective cohort of unselected epilepsy patients, but clinical evaluation of our model was not the main objective of this work. In addition, our ground truth was the visual assessment from the nuclear medicine physician using the standard PET which is an inherent limit to show the potential of the P-HQ PET. It would be interesting to evaluate our method in patients for which the standard quality PET was negative, but this is a very restrictive subpopulation where “ground truth” is often impossible to obtain as patients then neither undergo depth-electrode investigations nor surgery. Nevertheless, the patient with a small right post-central hypometabolism (Figure 8) underlines P-HQ PET's potential for clinical application. This work can be put into perspective with the work of Baete and Goffin (12, 61) that used the anatomy-based maximum a-posteriori (A-MAP) reconstruction algorithm to improve detection of small areas of cortical hypometabolism. Their method showed promise to increase detectability of hypometabolic areas on interictal [^18^F]FDG PET in a cohort of 14 patients with FCD. FCDs are the most commonly resected epileptogenic lesions in children and the third most common lesions in adults (8). FCD type II is a malformation with disrupted cortical lamination and specific cytological abnormalities (62). Surgery remains the treatment of choice in drug resistant patients and relies on lesion localization (63). In Goffin et al. (12) improvement failed to reach significance due to the small sample size, but underlined the clinical potential of such methods. For epilepsy surgery, the outcome of seizures and long-term results, including discontinuation of antiepileptic drugs, is highly dependent on the discovery of an epileptogenic lesion in the surgical specimen; for example, for FCD the chance of being seizure-free increases to 67% for positive sample (64). Imaging has an important role to localize FCD (9) in particular [^18^F]FDG PET (4, 65). In a study assessing the impact of imaging on FCD surgery outcome, there was no significant difference between FCDs detected on [^18^F]FDG PET, whether MRI had been positive or negative (66).

We quantitatively and qualitatively validated our model on simulated data with and without epilepsy-typical lesions. We also illustrated its potential applicability to clinical data. The next step is to assess the performance of the P-HQ PET in a clinical study, ideally in a large cohort of patients with well-localized lesions (FCDs), such as seizure-free subjects after brain surgery It will be also important to evaluate performance of nuclear medicine physicians with different levels of experience: P-HQ PET should be seen as a diagnostic support to improve reader detection and confidence, allowing non-expert readers to perform closer to expert reader performance. Another interesting perspective would be to assess the improvement of an AI based anomaly detection model (67) with the P-HQ PET compared to the standard PET.

## Conclusion

In this work, we trained a deep learning model to map S-SQ PET to their GT-HQ PET using a new large realistic Monte-Carlo simulated database. In an independent test set, the P-HQ PET showed improved image quality compared to S-SQ PET across several quantitative objective metrics. Moreover, in the context of epilepsy simulated lesions, the P-HQ PET improved the relative lesion activity and their visual detection. Following this validation on simulated lesion data and the successful clinical application to illustrate the proof-of-concept that a model trained on Monte-Carlo simulated PET data is applicable on real data, next steps are to perform a generalization study and to assess the performance of the P-HQ PET in a cohort of epilepsy patients with well-characterized lesions and/or normal standard-quality PET.

## Data availability statement

MRI data used are available at https://sites.google.com/view/calgary-campinas-dataset/download?authuser=0. The MRxFDG data used are available from the corresponding author at https://doi.org/10.1186/s13550-021-00830-6. A sample of standard and high quality pet data are available at https://osf.io/4j6xu and further access can be requested by contacting the corresponding author. Comparable deep learning model code are available at https://github.com/Project-MONAI/MONAI.

## Ethics statement

The studies involving human participants were reviewed and approved by King's College London and Guy's and St Thomas' PET Center, St Thomas' Hospital, London (Ethics Approval: 15/LO/0895). Written informed consent to participate in this study was provided by the participants' legal guardian/next of kin. Written informed consent was obtained from the individual(s), and minor(s)' legal guardian/next of kin, for the publication of any potentially identifiable images or data included in this article.

## Author contributions

AF designed the study, implemented the deep learning method, analyzed the data, and wrote the manuscript draft. NL performed a visual analysis of the PET images. TD, NC, AF, and IM performed the data simulations and image reconstructions. AR provided expertise for the simulation and network implementation. TG provided expertise with the network training. AH co-wrote the second manuscript draft. CM contributed to the acquisition and reconstruction of clinical PET data. AH, NC, and CL guided and supervised the project. All authors contributed to critically reviewing and approving the manuscript and read and approved the final manuscript.

## Funding

This research was funded in whole, or in part, by the Wellcome Trust [WT 203148/Z/16/Z]. For the purpose of open access, the author has applied a CC BY public copyright licence to any Author Accepted Manuscript version arising from this submission. AF received funding from the French branch of the International League Against Epilepsy (ILAE), Ligue Fran7aise contre l'Epilepsie (LFCE), the Labex Primes from Lyon University, Lyon and Hospices Civils de Lyon.

## Conflict of interest

The authors declare that the research was conducted in the absence of any commercial or financial relationships that could be construed as a potential conflict of interest.

## Publisher's note

All claims expressed in this article are solely those of the authors and do not necessarily represent those of their affiliated organizations, or those of the publisher, the editors and the reviewers. Any product that may be evaluated in this article, or claim that may be made by its manufacturer, is not guaranteed or endorsed by the publisher.
