# Supplementary material for: PET image enhancement using artificial intelligence for better characterization of epilepsy lesions
Source: Front Med (Lausanne). 2022 Nov 16;9:1042706. doi: 10.3389/fmed.2022.1042706 (PMC9708713; doi:10.3389/fmed.2022.1042706)
Supplement: Supplementary file 1 [file Data_Sheet_1.docx]

Supplementary Material

**1. Model comparison**

We added the comparison in the supplementary data section. We compared a UNet model from the Monai Project (<https://docs.monai.io/en/stable/networks.html#unet>) with the selected architecture ResNet. The UNet was a 5-layer network with down/upsampling by a factor of two at each layer (channels=16, 32, 64, 128, 256) with one convolution residual units with a kernel of 3x3x3 convolutions, each followed by a batch normalization and PReLU activation. Predicted high-quality (P-HQ) PETs from both models are shown in Supplementary Figure 1. The metrics were close (Supplementary Table 1) but visual inspection showed that the UNet output was blurrier with a discrete checkerboard artifact.

**Supplementary Table 1**: Mean and standard deviation of the root mean squared error (RMSE), peak signal to noise ratio (PSNR) and structural similarity index measure (SSIM) for high quality (HQ) PET images in the test set for the ResNet and the UNet. The comparator is the ground-truth HQ PET.

|  | Root mean squared error | Peak signal-to-noise ratio (dB) | Structural Similarity index measure |
| --- | --- | --- | --- |
| ResNet | 1359 ± 888 | 21.8 ± 1.8 | 0.929 ± 0.011 |
| UNet | 1464 ± 947 | 21.1 ± 1.9 | 0.920 ± 0.009 |


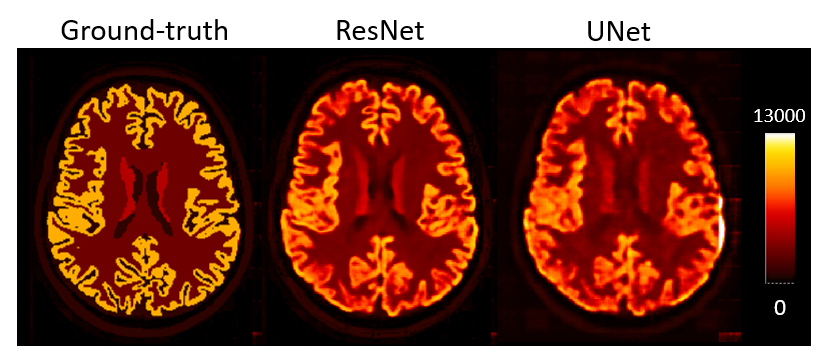


**Supplementary Figure 1**: Results from one subject belonging to the test dataset. The first column depicts the Ground Truth High Quality (HQ) PET, the second column the Predicted HQ PET from the ResNet (the proposed network) and the third column the Predicted HQ PET from the UNet.

**2. Recovery analysis per activity ratio**

In the Supplementary figure 2 and 3, the grey matter (GM) and white matter (WM) recovery coefficient (RC) were computed in the simulated standard quality (S-SQ) PET and predicted high-quality (P-HQ) PET according to the ground truth (GT) GM/WM ratio. One can see that that WM RCs of S-SQ PET ranged from 0.45 to 0.52 and did not highly depend on the actual GM/WM ratio. On the contrary, GM RC of S-SQ PET were lower (from 0.26 to 0.36) and did depend on actual GM/WM thresholds. This showed that GM was much more affected by partial volume effect than WM, expected given the complex circumvoluted pattern of GM and small thickness of cortex relative to system resolution, whereas eroded WM mask has much larger homogeneous areas and very little spill-in and spill-out. Thus the method improved the recovery coefficient for both the GM and the WM, but to a different extent because the network task of recovering the ground truth value in the GM region is more challenging. The mean GM recovery was 0.79 ± 0.04 versus 1 ± 0.05 for the white matter in S-SQ PET.


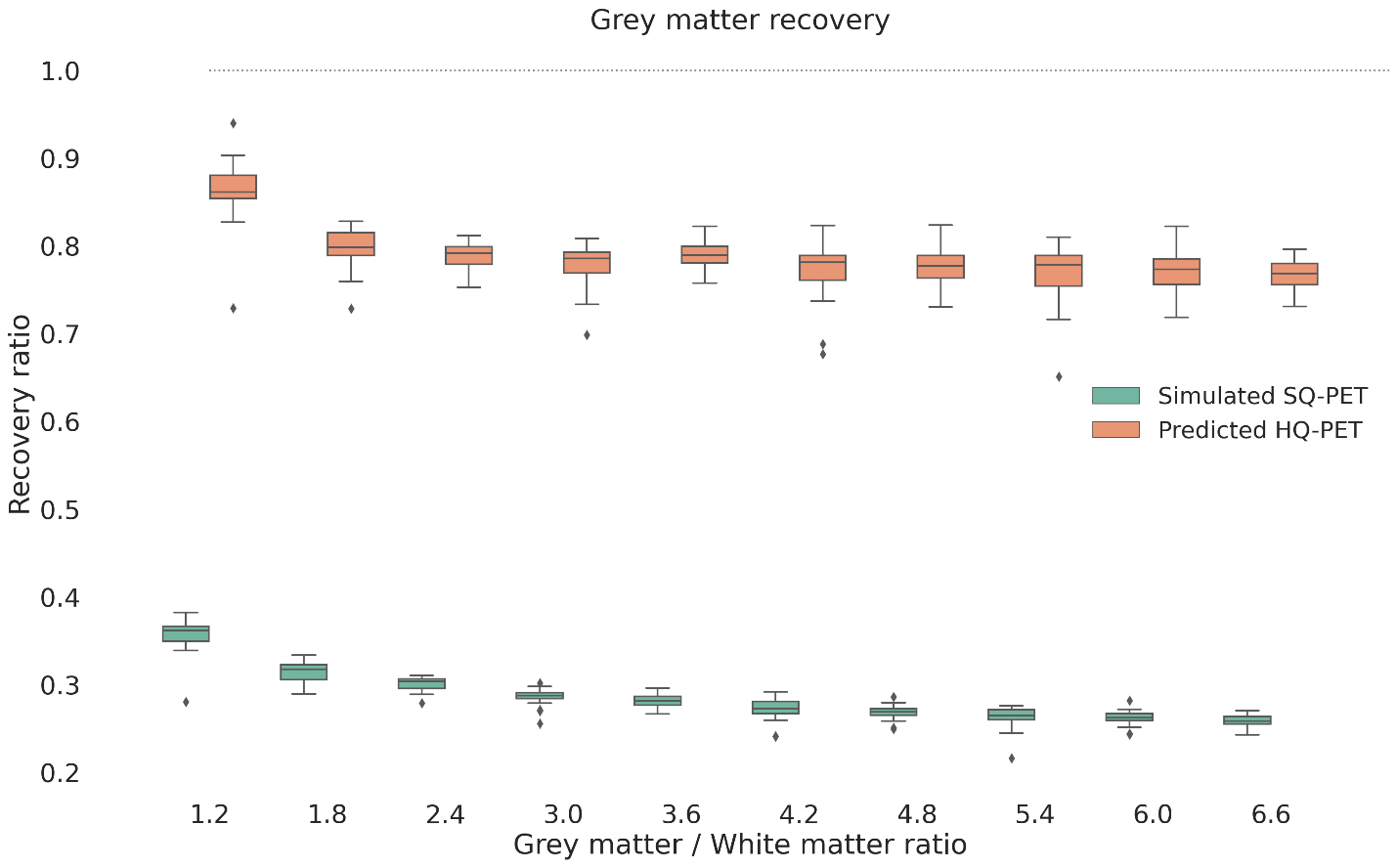


**Supplementary figure 2**: Recovery coefficient for the grey matter (GM) in the test set shown for each GM/WM ratio for the simulated standard-quality (SQ) PET (green) and the predicted high-quality -HQ) PET (orange). Dotted line for a recovery ratio of 1.


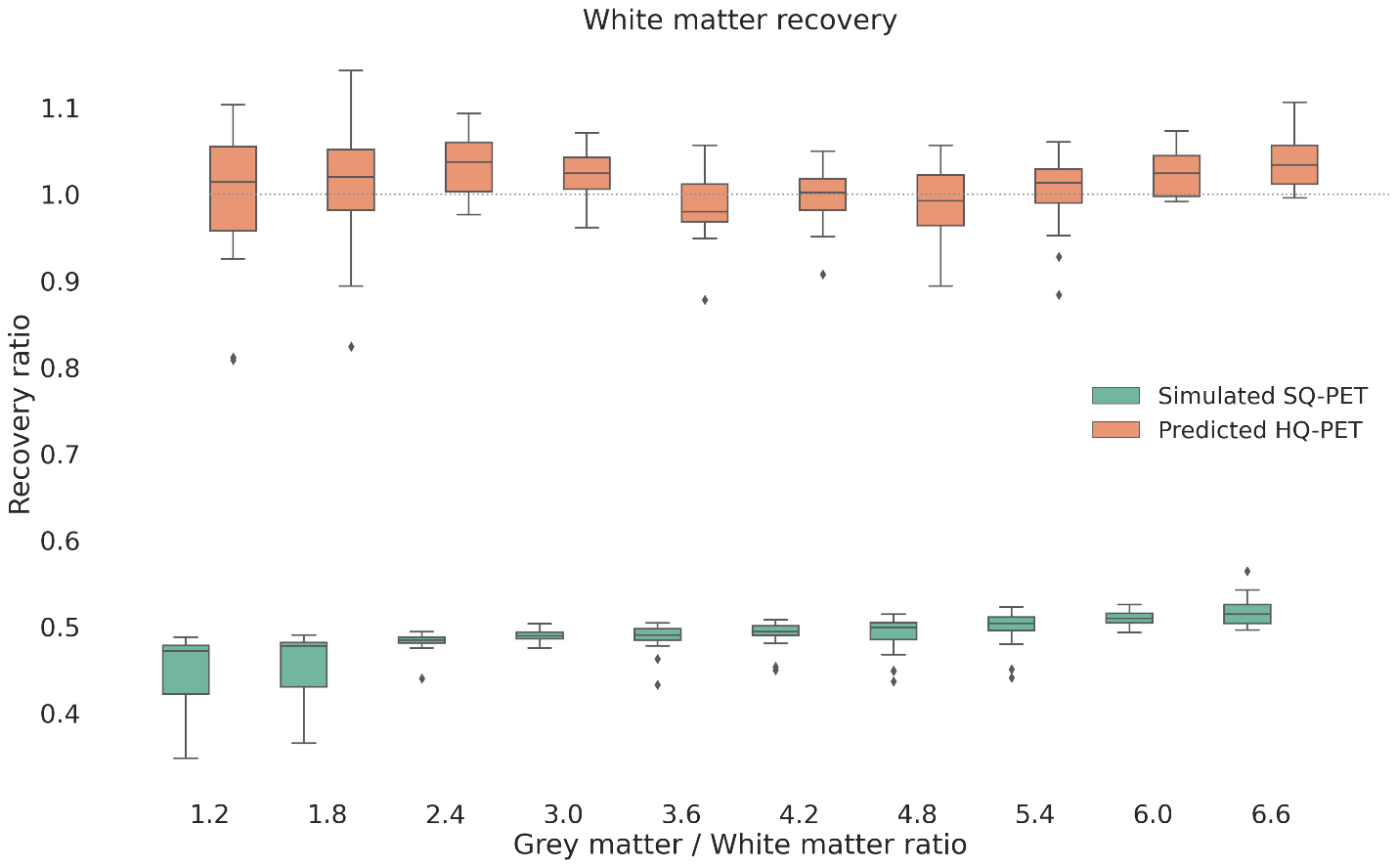


**Supplementary figure 3**: Recovery coefficient for the eroded WM in the test set presented for each GM/WM ratio for the simulated standard-quality (SQ) PET (green) and the predicted high-quality (HQ) PET (orange). Dotted line for a recovery ratio of 1.
